# Prevalence of neural tube defects at Debre Berhan Referral Hospital, North Shewa, Ethiopia. A hospital based retrospective cross-section study

**DOI:** 10.1371/journal.pone.0261177

**Published:** 2022-02-02

**Authors:** Zerihun Kindie, Abay Mulu

**Affiliations:** 1 Department of Anatomy, School of Medicine, College of Health Sciences, Assosa University, Assosa, Ethiopia; 2 Department of Anatomy, School of Medicine, College of Health Sciences, Addis Ababa University, Addis Ababa, Ethiopia; Ohio State University, UNITED STATES

## Abstract

**Background:**

Neural tube defect (NTD) is a structural defect of the central nervous system of the developing embryo during the first month of developmental process. Most congenital malformations are potentially preventable cause of perinatal morbidity and mortality. Worldwide, around 10% of infant mortalities are due to nervous system defects. In Ethiopia there are limited published data regarding the prevalence and established preventive strategy of NTDs. The purpose of this study was to assess the prevalence of NTDs among pregnancy outcomes in Debre Berhan Referral Hospital (DBRH), Ethiopia.

**Methods:**

Hospital based retrospective cross sectional, descriptive study was conducted on registration of all pregnancy outcomes from August 30, 2017 to August 30, 2019 at DBRH, Ethiopia. The collected data were checked for completeness and consistencies, and cleaned, coded and entered using Epi data version 4.2 and exported to Statistical Package for Social Sciences (SPSS) software version 20 for analysis. Variables were interpreted per 1000 pregnancies and those variables having p<0.05 was considered as statistically significant.

**Results:**

The total prevalence of NTDs was 10.9 (95% CI 8.9 to 13.3) per 1000 pregnancies and the prevalence of each NTD type was anencephaly 5.6 (95% CI 4.2 to 7.4) per 1000 pregnancies, spina bifida 3.5 (95% CI 2.4 to 4.9) per 1000 pregnancies, encephalocele 1.1 (95% CI 0.6 to 2.0) per 1000 pregnancies, and both spina bifida and anencephaly 0.7(95% CI 0.3 to 1.4) per 1000 pregnancies. Among livebirths, aborted, stillbirths and medically terminated pregnancies (n = 8862), there were 50 anencephaly cases, 31 spina bifida cases, 10 encephalocele cases, and 6 cases affected by both spina bifida and anencephaly.

**Conclusion:**

The prevalence of NTDs in this study was among the highest globally reported. The total prevalence was 10.9 per 1000 pregnancies. Increased periconceptional folic acid use, counseling for women with certain medical illnesses at higher risk for NTDs, and early maternal screening for genetic factors are possible approaches to reduce in NTDs in the population.

## Background

Neural tube defects (NTDs) are structural defects of the central nervous system that affects the brain, spine and spinal column of the developing embryo during the first month of developmental process. Most common congenital malformations are potentially preventable cause of perinatal morbidity and mortality [1]. Among all NTDs spinal bifida and anencephaly are the two most common forms [2]. Worldwide, around 10% of infant mortalities are due to nervous system defects [3]. The most common NTD cases are anencephaly and spina bifida; however, anencephaly is a fatal NTD type, but babies with spina bifida often survive following surgical interventions [4]. One scientific survey from eighteen countries in six World Health Organization (WHO) regions related that the prevalence of the NTDs based on livebirths to be 1.67/1000 births for total NTD prevalence [5]. However, the incidence of neural tube defects (NTDs) is coming to decline in recent years in industrialized countries, while it still remains high in the less developed countries of Latin America, Africa, the Middle East and Far East Asia [6]. It is estimated that approximately 300,000 babies are born each year with NTDs worldwide [7].

Studies conducted at the Texas-Mexico border noted higher occurrence of NTDs among women with folic acid deficiency, B12 deficiency, obesity, or diabetes [8]. Some evidence suggest that presence of modern technology which leads to early detection and termination of NTDs, improvement of folic acid supplementation and better socioeconomic status/living standard leads to reduction in prevalence of NTDs worldwide [9].

Unlike developing countries, including Ethiopia, the identification of the risk factors (maternal nutritional deficiency, chemical exposure, medical and fever illness and life style) in decreasing the prevalence of NTDs is well established in the developed world [10]. Studies about prevalence of NTDs in Ethiopia are scarce in different regions of the country. The main aim of this study was to collect information about the prevalence of NTDs in the North Shewa region to estimate overall prevalence at the country level.

## Methods and materials

### Study setting and study population

Retrospective cross sectional medical chart review study design was conducted from September 01 to October 30, 2019 on medical delivery charts from August 30, 2017 to August 30, 2019 at Gynecological and Obstetrics ward of Debre Berhan Referral Hospital, North Shewa, Ethiopia. This hospital was selected purposely based on the availability of patients from all nearby regions of the country as it is one of the referral and specialized teaching hospital in Ethiopia that gives services for North Shewa residents. The hospital is the only referral hospital in the region in which all neural tube defect cases are referred first to it before referred to the capital city of Ethiopia. Sample size of the study was calculated considering the prevalence of NTDs to 50 cases per 1000 pregnancy outcomes due to absence of similar research in the study area and using a single proportion formula at 95% CI and 2.5% margin of error, a total of 1537 minimum sample was calculated, but due to rare case 8862 medical delivery charts were conveniently revised in the study periods. Cases with any ambiguity or multiple congenital anomalies, gestational age of medically terminated fetus <12 weeks and congenital anomalies other than NTDs were excluded. Because, in the developmental processes, presences of some congenital anomalies are complicated with neural tube defects, and the preceding of neural tube defects are also complicated with other congenital anomalies. Therefore, it is very difficult to differentiate which one comes first, that is why such cases are excluded. Gross identification of neural tube defects in all terminated pregnancies <12 weeks of gestation is difficult and all these cases were also excluded, regardless of whether a NTDs were suspected.

### Data collection

Medical delivery charts from August 30, 2017 to August 30, 2019 were revised based on well-structured and pretested questionnaire through trained BSc midwives for the prevalence of NTDs. Medical charts were selected anonymously from the total pooled samples. To fully anonymize the data, we gave sequential numbers for all the medical charts that fulfill inclusion criteria and took every fixed interval samples after randomly taking the first sample. Before data collection, pretest was done in 5% of the sample size population at Wollo Referral Hospital that is another regional hospital located in South Wollo at the same level as Debre Berhan referral hospital, which was not included in the study area. The aim of the pretest was to make necessary adjustments on the study tool before the actual data collection began.

### Data analysis and processing

The data were checked for completeness and consistencies, cleaned, coded and entered using Epi data version 4.2 and exported to Statistical Package for Social Sciences (SPSS) software version 20 for analysis. The results are presented in tables and figures.

### Ethics approval and consent to participate

Ethical clearance was obtained from the Department Research Ethics Review Committee (DRERC), Institutional Review Board (IRB), Addis Ababa University, and Department of Anatomy (ANA/0015/2011, on April 16, 2019). Formal letter or clearance was sent to the Department of Gynecological and Obstetrics of Debre Berhan Referral Hospital to get consent for data collection. Then permission was taken from hospitals higher management and data were collected. The Department Research Ethics Review Committee waived any requirement for ethical issues. The cooperating hospitals had the responsibility of obtaining informed consent from their patients for using the medical chart information, while maintaining confidentiality, for the purpose of research, with the justification that the findings would benefit the community.

## Result

### Prevalence of neural tube defects

During the study period, a total of 8,862 medical charts (out of them, 7920 were delivery, 722 were abortion and 220 were medically terminated) of pregnancy after the 12th week of gestation were assessed. The term abortion in this study refers spontaneous abortions. From those, 97 pregnancies (out of case of NTDs, 60 cases from delivery, 26 cases from abortion and 11 cases from medically terminated) were affected by NTDs.

Among livebirths, aborted, stillbirths and medically terminated cases, the birth prevalence of NTDs was 10.9 (95% CI 8.9 to 13.3) per 1000 pregnancies. Including medically terminated cases, 50 cases per 8862 were anencephaly, 31 cases per 8862 were spina bifida, 10 cases were encephalocele and 6 cases per 8862 were both spina bifida and anencephaly.

Prevalence of anencephaly was 5.6 (95% CI 4.2 to 7.4) per 1000 pregnancies, spina bifida was 3.5 (95% CI 2.4 to 4.9) per 1000 pregnancies, encephalocele was 1.1 (95% CI 0.6 to 2.0) per 1000 pregnancies and both spina bifida and anencephaly was 0.7 (95% CI 0.3 to 1.4) per 1000 pregnancies.

**Fig 1:**
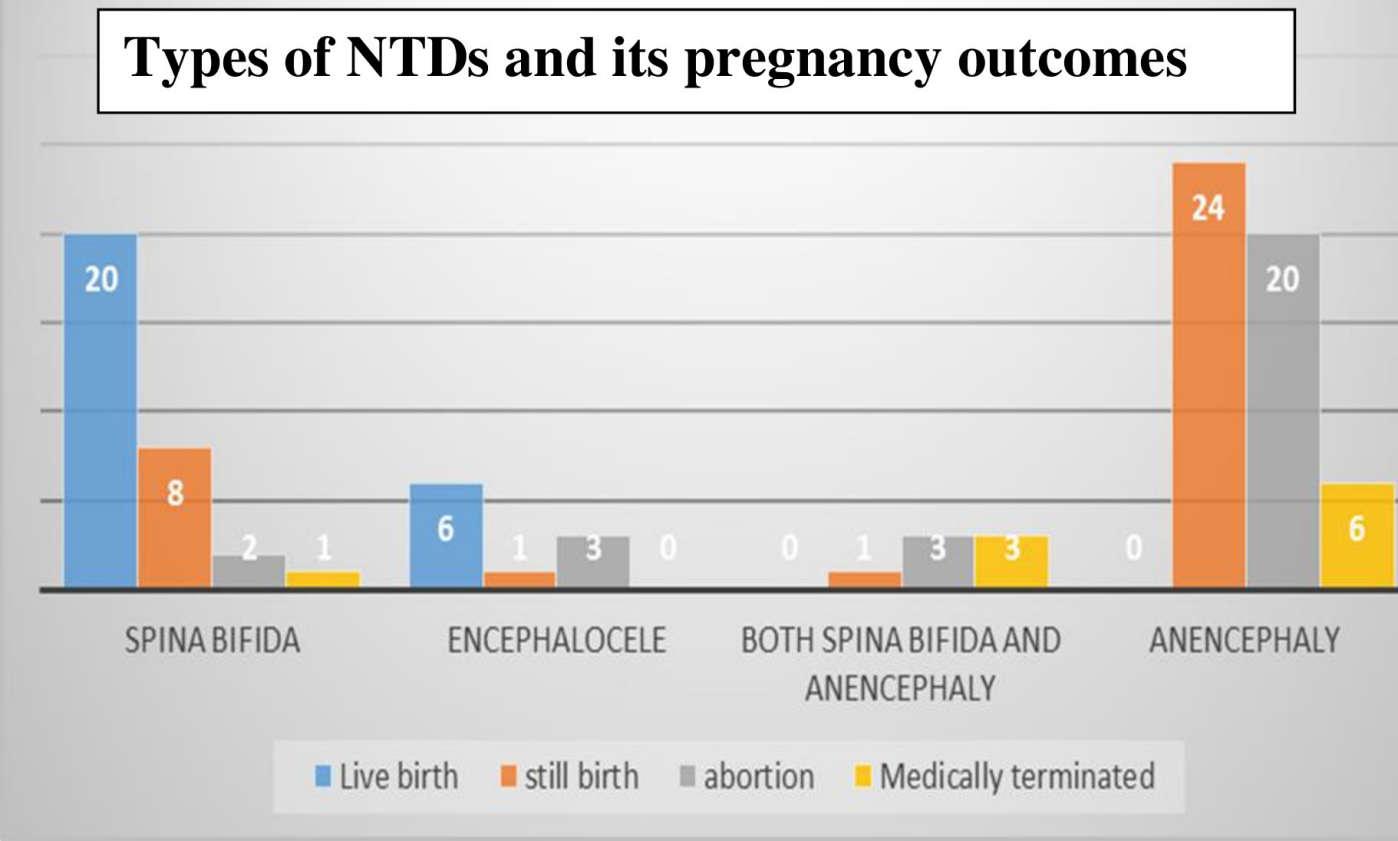
Types of NTDs and its pregnancy outcomes at Debre Berhan Referral Hospital, from August 30, 2017 to August 30, 2019 (*n* = 97). Stillbirth is delivery of dead fetus after 20^th^ week of development. Abortion is ending of pregnancy before 20^th^ week of development. Medically terminated means ending of pregnancy for medical reasons.

In this study, cases of anencephaly were the most common types of the NTDs. Among types of NTDs most of anencephaly cases were aborted or stillbirths, whereas spina bifida and encephalocele cases were more likely to be liveborn. [Fig 1].

From a total of 97 cases, maternal occupation was house wife 21.6% (21/97) and farmer 28.9% (28/97). From 97 NTDs case, 87.6% (85/97) and 95.6% (93/97) had not taken folic acid prior to conception or during periconceptional period and were not taken folic acid at any time, respectively. Periconceptional period in this study refers the period three months before the occurrence of pregnancy.

Multiparous and primiparous were the commonest gravidities, each accounting for 71% (69/97) and 16.5% (16/97), respectively. Most of NTDs, 33% (32/97), 23.7% (23/97) and 16.5% (16/97) gestational age were 37–40 weeks, 32–36 weeks and <28 weeks, respectively.

As shown in Table 1; when comparing types of NTDs by gender, 74% (37/50) anencephaly were males, whereas 67.7% (21/31) spina bifida were females.

**Table 1:** Types of NTDs and Genders at Debre Berhan Referral Hospital, North Shewa, Ethiopia, 2019. [N = 97].

		Types of NTD				Total
		Anencephaly	Spina bifida	Encephalocele	Both anencephaly and spinal bifida	
**Gender**	**Male**	37	10	3	1	51
	**Female**	13	21	7	5	46
**Total**		50	31	10	6	97

Majority of the mothers, 47.4% (46/97) had no antenatal care (ANC) follow up and 28.9% (28/97) were start ANC follow up after start of the 3^rd^ trimester. From 97 NTDs, 31.9% (31/97) spina bifida and 22.7% (22/97) anencephaly occurs among multiparous mothers and 16.5% (16/97) and 12.4% (12/97) anencephaly occurs from primiparous and nulliparous, respectively. [Table 2].

**Table 2:** Types of NTDs and Parity at Debre Berhan Referral Hospital, North Shewa, Ethiopia, 2019. [N = 97].

		Parity			Total
		Nulliparous	Primiparous	Multiparous	
**Types of NTD**	**Anencephaly**	12	16	22	50
	**Spina bifida**	0	0	31	31
	**Encephalocele**	0	0	10	10
	**Both anencephaly and spina bifida**	0	0	6	6
**Total**		12	16	69	97

Anencephaly of 20.6% (20/97) and 16.5% (16/97) mainly presented with in gestation age of 32–36 weeks and <28 weeks, respectively. [Table 3].

**Table 3:** Types of NTDs and gestational age at Debre Berhan Referral Hospital, North Shewa, Ethiopia, 2019. [N = 97].

		Gestational age of NTD					Total
		<28 weeks	28–31 weeks	32–36 weeks	37–40 weeks	>40 weeks	
**Types of NTD**	**Anencephaly**	16	14	20	0	0	50
	**Spina bifida**	0	0	3	28	0	31
	**Encephalocele**	0	0	0	4	6	10
	**Both anencephaly and spina bifida**	0	0	0	0	6	6
**Total**		16	14	23	32	12	97

## Discussion

Hospital based retrospective cross sectional study design was conducted in this study. The study consists of reviewing 8862 pregnant mothers’ medical charts; among those, 97 pregnancies were with NTDs. Birth defects are major causes of mortalities before five years of age and Neural tube defects (NTDs) are one of the most common major birth defects next to congenital heart diseases [11]. In this study, the total prevalence for all types of NTDs was found to be 10.9 per 1000 pregnancies, which is six times more prevalent than the study done in six countries by World Health Organization (WHO) 1.67/1000 [5], and three times more prevalent than a study done in Sudan 3.48/1000 [12], and 1.8 times more prevalent than a study done in three teaching hospitals in Addis Ababa 6.1/1000 [13]. But this prevalence was less than the prevalence of NTDs in Tigray region 13.8/1000 [10].

The specific finding of anencephaly (5.6/1000) is higher than prevalence of NTDs reported in Africa and Ethiopia, and much more higher than reports from six World Health Organization study sites in Africa 0.25/1000 [4], Malawi 3.1/1000 [14], and in three teaching hospital Addis Ababa 4.2/1000 [13]. With respect to other types of NTDs, this study had higher prevalence of spinal bifida (3.5/1000) compared to six World Health Organization study sites in Africa 1.13/1000 [4], Malawi 0.47/1000 [9] and Cape Town 1.74/1000 [14], except in Tigray region 6.4/1000 [15]. NTDs were observed to occur almost equally among males (52.6%) and females (47.4%), but anencephaly cases were more likely to be male (74%) and spina bifida cases were more likely to be female (67.7%), which is not comparable to reports from a case-control study based on the Oxford Record Linkage about 70 percent of the children with anencephaly and 60 percent of the children with spina bifida were females [16].

Adequate surveillance data are needed to develop effective prevention strategies. The high prevalence of this study might be as a results of nutritional factors, family history of NTDs, lack of routine folic acid supplementation and absence of folic acid fortification programs. In this study, there was no periconceptional folic acid supplementation for 90% of the case mothers, which is in line with a study conducted in Italy [17] and similar to findings of studies conducted in Algeria [18], Addis Ababa [13] and Tigray [15], where 86%, 92.2% and 85.3% of case mothers, respectively, did not take folic acid. Another study found out more cranial neural tube defects in females in relation to X-chromosome factor affecting neural folding processes [19], and ratio change is reported after fortification with folic acid [20].

### Conclusion

In conclusion, the prevalence of neural tube defects in this study is among the highest globally reported (Africa, Europe, America, and many Asian countries as well as reported from Addis Ababa Hospitals). Results indicated that the most prevalent NTD being anencephaly and spina bifida. Increased periconceptional folic acid use, counseling for women with certain medical illnesses at higher risk for NTDs, and early maternal screening for genetic factors are possible approaches to reduce in NTDs in the population.

## Limitations of the study

Our comments regarding possible risk factors and prevention efforts are speculations based on prior studies in comparison to patterns observed among the cases in our data. We are unable to draw more definitive conclusions, as we do not report the distributions among the unaffected pregnancies in this population for comparison.

We are unable to screen NTDs before 12 weeks of gestation and prevalence may be affected due to the absence of these group.
